# Association Between Neuroticism and Risk of Lung Cancer: Results From Observational and Mendelian Randomization Analyses

**DOI:** 10.3389/fonc.2022.836159

**Published:** 2022-02-14

**Authors:** Xiaoxia Wei, Xiangxiang Jiang, Xu Zhang, Xikang Fan, Mengmeng Ji, Yanqian Huang, Jing Xu, Rong Yin, Yuzhuo Wang, Meng Zhu, Lingbin Du, Juncheng Dai, Guangfu Jin, Lin Xu, Zhibin Hu, Dong Hang, Hongxia Ma

**Affiliations:** ^1^ Department of Epidemiology, Center for Global Health, School of Public Health, Nanjing Medical University, Nanjing, China; ^2^ Department of Thoracic Surgery, The First Affiliated Hospital of Nanjing Medical University, Nanjing, China; ^3^ Department of Thoracic Surgery, Jiangsu Key Laboratory of Molecular and Translational Cancer Research, Jiangsu Cancer Hospital, Jiangsu Institute of Cancer Research, The Affiliated Cancer Hospital of Nanjing Medical University, Nanjing, China; ^4^ Jiangsu Key Lab of Cancer Biomarkers, Prevention and Treatment, Collaborative Innovation Center for Cancer Personalized Medicine, Nanjing Medical University, Nanjing, China; ^5^ Department of Cancer Prevention, The Cancer Hospital of the University of Chinese Academy of Sciences (Zhejiang Cancer Hospital), Hangzhou, China; ^6^ Institute of Basic Medicine and Cancer (IBMC), Chinese Academy of Sciences, Hangzhou, China; ^7^ Research Units of Cohort Study on Cardiovascular Diseases and Cancers, Chinese Academy of Medical Sciences, Beijing, China

**Keywords:** lung cancer, neuroticism, genetic risk, prospective analysis, Mendelian randomization study

## Abstract

**Background:**

It remains undetermined whether neuroticism affects the risk of lung cancer. Therefore, we performed complementary observational and Mendelian randomization (MR) analyses to investigate the association between neuroticism and lung cancer risk.

**Methods:**

We included 364,451 UK Biobank participants free of cancer at baseline. Neuroticism was ascertained using the 12-item of Eysenck Personality Inventory Neuroticism Scale. Multivariable Cox regression models were used to calculate hazard ratios (HRs) and 95% confidence intervals (CIs). Two-sample MR analysis was carried out with summary genetic data from UK Biobank (374,323 individuals) and International Lung Cancer Consortium (29,266 lung cancer cases and 56,450 controls). Furthermore, we calculated a polygenic risk score of lung cancer, and examined the joint-effect and interaction between neuroticism and genetic susceptibility on lung cancer risk.

**Results:**

During a median follow-up of 7.13 years, 1573 lung cancer cases were documented. After adjusting for smoking and other confounders, higher neuroticism was associated with an increased risk of lung cancer (HR _per 1 SD_=1.07, 95% CI: 1.02-1.12). Consistently, MR analysis suggested a causal effect of neuroticism on lung cancer risk (OR _IVW_=1.10, 95% CI: 1.03-1.17). Compared to individuals with low neuroticism and low PRS, those with both high neuroticism and high PRS had the greatest risk of lung cancer (HR=1.82, 95%CI: 1.51-2.20). Furthermore, there was a positive additive but no multiplicative interaction between neuroticism and genetic risk.

**Conclusions:**

Our findings suggest that neuroticism is associated with an elevated risk of incident lung cancer, which is strengthened by the genetic susceptibility to lung cancer. Further studies are necessary to elucidate underlying mechanisms.

## Introduction

Neuroticism is a personality trait that reflects relative stability to experience negative emotions. Neuroticism is one of the major dimensions of the Five-Factor Model of personality (1), and it is also one of the most studied psychological dispositions because of its relevance ranging from normal to abnormal emotional functioning (2). Individuals with higher neuroticism would be more likely to be worried, anxious and emotionally unstable (3). There is growing evidence that neuroticism is associated with a wide range of adverse health outcomes, including the occurrence of mental disorders (4), diabetes (5), cardiovascular disease (CVD) (6), cancer (7, 8), and mortality (9). The underlying mechanisms may be directly or indirectly related to endocrine, inflammation, and harmful behaviors (e.g., cigarette smoking and alcohol abuse) (10, 11).

Evidence linking regarding neuroticism to lung cancer risk, however, is scarce and inconsistent. For instance, several retrospective case-control studies have suggested that a low degree of neuroticism was significantly associated with an increased risk of lung cancer (7, 12), while prospective studies found either null (13–17) or positive associations (18) between higher neuroticism and lung cancer risk. Notably, most of the previous studies were limited by the lack of strict adjustment for confounding variables (e.g., smoking and alcohol use), a short follow-up period or small numbers of lung cancer cases. Thus, the exact association between neuroticism and lung cancer still needs to be determined in well-designed prospective studies with large samples.

Moreover, in contrast to observational studies, which are susceptible to reverse causation and confounding bias, Mendelian randomization (MR) analysis is an established approach to assess the causal effect of an exposure on an outcome by using genetic variants as a proxy for the exposure (19). Genetic variants are randomly assigned at gametogenesis, independent of environmental factors and unaffected by disease processes, thus can minimize the influence of residual confounding and reverse causation (20). This approach has been successfully applied to verify different etiological associations (21–24), such as low education is a causal risk factor in the development of lung cancer.

Herein, we performed complementary observational and Mendelian randomization (MR) analyses to assess the association between neuroticism and lung cancer risk based on the UK Biobank resource. In addition, because previous studies, including ours (25, 26), suggest that the genetic factors may modify the associations between behavioral/environmental risk factors and lung cancer risk; therefore, we further assessed the potential joint and interactive effect between neuroticism and genetic susceptibility represented by a polygenic risk score (PRS) on lung cancer risk.

## Subjects and Methods

### Study Design and Participants

The UK Biobank is a large population-based prospective cohort study, with the study design and data acquisition process described in detail previously (27). Briefly, over 500,000 people aged 40-69, were enrolled between 2006 and 2010 *via* 22 health assessment centers across England, Wales and Scotland. Social demographics, lifestyle, health-related information are obtained through touch-screen questionnaires and physical measurements. Blood, saliva, and urine were also collected from each participant. The UK Biobank has full ethical approval from the North West Multi-center Research Ethics Committee. All participants provided written informed consent at recruitment.

In the current study, we excluded participants with prevalent cancer at recruitment (n=46,533) and missing data on neuroticism (n=91,523), leaving 364,451 participants in the observational analysis. In addition, only 300,465 individuals of European descent were available for the genetic analysis (**Supplementary Figure S1**).

### Exposure and Covariate Ascertainment

In the baseline survey, neuroticism was assessed using the 12-item neuroticism subscale from the Eysenck Personality Inventory Neuroticism Scale (EPIN‐R) (28). Responses to each item were either “Yes” or “No”, which were summed to produce a total score that varied from 0 to 12. A higher score indicated greater neuroticism. Other covariate data were collected at baseline using standard protocols, including socioeconomic characteristics (age, sex, ethnic background and education level), health-related factor (family history of lung cancer), and lifestyle factors (smoking, alcohol intake, body mass index and physical activity).

### Outcome Ascertainment

Incident cases of lung cancer within the UK Biobank cohort were identified through linkage to national cancer registries in England, Wales and Scotland. Participants were followed up to date of diagnosis of lung cancer, date of withdrawal from the study, date of death or loss follow-up (referring to March 31, 2016, for England and Wales and October 31, 2015, for Scotland), whichever came first. Incident lung cancer was identified using the International Classification of Diseases, Tenth Revision codes of C33 and C34.

### Mendelian Randomization Analyses

We conducted two-sample Mendelian randomization (MR) analyses to assess the causal association between neuroticism and lung cancer risk. Instrumental variables for neuroticism were selected from a Genome-wide association study (GWAS) of 374,323 individuals of European ancestry in the UK Biobank (GWAS ID: ukb-b-4630), available in the IEU GWAS database (https://gwas.mrcieu.ac.uk/) (29). Among the genome-wide-significant SNPs (*P*<5×10^-8^) identified in neuroticism GWAS, we obtained 116 independent SNPs after exclusion of correlated SNPs based on a linkage disequilibrium level of r^2^ <0.01 (**Supplementary Table S1**). Moreover, SNPs with intermediate allele frequencies (MAF >0.42) were considered to be strand-ambiguous and removed from the analysis. Consequently, the instruments for neuroticism explained 1.26% (F-statistic 40.63). Besides, summary-level data for lung cancer was obtained from the International Lung Cancer Consortium (totalling 29,266 lung cancer cases and 56,450 controls; dbGAP: phs000876) (30).

The primary method in MR analyses was the inverse-variance weighted (IVW) (31), followed by sensitivity analyses using the maximum likelihood (ML) methods (32), weighted median (WM) (33), MR-Egger regression (34) and MR Robust Adjusted Profile Score (RAPS) (35). We further applied two diagnostic tests, including the MR Egger intercept test of significant deviation from the null (36), and Cochran’s Q-statistic to assess heterogeneity. Moreover, to rule out possible pleiotropic effects, we examined the genetic instruments in the Phenoscanner GWAS database (http://www.phenoscanner.medschl.cam.ac.uk/) to assess previously reported associations (*P <*5 × 10^−8^) with confounders (smoking or BMI), and then assessed the effects after manual filtering the related SNPs from the MR analyses. Finally, we performed reverse-direction MR analyses to estimate the effect of lung cancer (exposure) on neuroticism. The MR analysis was performed using the TwoSampleMR R package.

### Polygenic Risk Score Calculation

The procedure for genotyping, imputation and quality control of the SNPs in the UK Biobank has been described elsewhere (37). In the present study, we created a polygenic risk score for lung cancer using 18 SNPs based on the largest available lung cancer GWAS of European descent (**Supplementary Table S2**) (30). Each SNP was recoded as 0, 1, or 2 according to the number of risk alleles; and then multiplied by its respective effect size (β-coefficient) of lung cancer to calculate the PRS: PRS =β_1_ × SNP_1_ + β_2_ × SNP_2_ +…+ β_n_ × SNP_n_ (38). The genetic risk was categorized into low (lowest tertile), intermediate (second tertile), and high (highest tertile) based on distributions of PRS in non-cases (38, 39).

### Other Statistical Analysis

Cox proportional hazards model was used to estimate the hazard ratio (HR) and corresponding 95% confidence interval (CI) for the association between neuroticism and incident lung cancer. The proportional hazards assumption was tested using Schoenfeld residuals. Neuroticism was modeled on the continuous (per 1-standard deviation [SD] increment) or quintile scale. Model 1 was adjusted for age at recruitment (continuous), sex, ethnic background (white, non-white), education (college or university degree, no degree) and family history of lung cancer (no, yes). In the multivariable model 2, we additionally adjusted for lifestyle factors, including smoking status (never, former, current <15, current ≥15 cigarettes/day, current amount unknown), alcohol intake frequency (daily or almost daily, three or four times a week, once or twice a week, once to three times a month, special occasions, never), BMI (kg/m^2^, <25, 25-29.9, ≥30), and physical activity (MET-h/week, <10, 10-50, ≥50). In genetic analyses, we further adjusted for the first ten genetic principal components and genotyping array batch. Missing data on covariates were coded as a missing indicator for categorical variables and with sex-specific median values for continuous variables. Statistical tests for trends were assessed by treating the variables continuously.

To assess the joint associations, we further classified participants into six groups according to PRS (low, intermediate, high) and neuroticism score (low, high), and estimated hazard ratios of incident lung cancer in different groups compared with those with low PRS and low neuroticism. To quantify the multiplicative interaction between neuroticism and genetic risk of lung cancer, we added an interaction term in the Cox proportional hazards regression models. Additive interaction was evaluated by relative excess risk due to interaction (RERI) and attributable proportion because of the interaction (AP), and its 95% confidence interval (CI) was calculated by drawing 1000 bootstrap samples from the estimation dataset (40). If there was additive interaction, the 95% CIs of the RERI and AP would not include 0.

In addition, to examine whether the associations between neuroticism and lung cancer risk differed by subgroups, stratified analyses were conducted by age at recruitment, sex, ethnic background, education, family history of lung cancer, smoking status, alcohol intake frequency, BMI, physical activity and histological subtypes. The heterogeneity of these stratified estimates was evaluated using the χ^2^ -based Cochrane’s Q test. Two sensitivity analyses were performed to assess the robustness of the findings: (1) excluding participants with less than one year of follow-up; and (2) using the Fine and Gray competing risk model (41) to account for potential bias due to the competing risks of death.

All above statistical analyses were performed with R (version 3.61) and *P* < 0.05 (two-sided) was considered statistically significant.

## Results

Of the 364,451 participants included in the analysis, 1573 developed incident lung cancer during a median follow-up period of 7.13 years. The baseline characteristics of the participants are shown in **Table 1**. Compared with those in the lowest quintile of neuroticism, participants in the highest quintile were younger, less educated, less physical activity, and more likely to be smokers and have higher BMI.

**Table 1:** Baseline characteristics of UK biobank participants by quintile of neuroticism.

Characteristic	Quintile categories of neuroticism				
	Q1 (0–1)	Q2 (2–3)	Q3 (4–5)	Q4 (6–7)	Q5 (8–12)
Participants (No.)	98157	80119	69178	53253	63744
Age at baseline (years) ^a^	56.96 (7.98)	56.61 (8.05)	56.02 (8.08)	55.52 (8.10)	54.74 (7.99)
Female, %	40.82	51.87	56.55	59.67	61.32
White race, %	94.70	95.22	95.36	95.49	94.88
College or university degree, %	37.35	34.88	32.82	31.16	28.9
BMI (kg/m^2^) ^a^	27.46 (4.49)	27.35 (4.61)	27.39 (4.81)	27.45 (4.92)	27.54 (5.16)
Physical activity (MET hour/week) ^a^	44.58 (43.62)	42.47 (41.47)	41.92 (41.85)	40.35 (40.52)	39.21 (40.54)
Family history of lung cancer, %	11.59	11.97	12.46	12.91	13.04
Alcohol intake frequency, %					
Never	7.03	6.85	7.12	7.43	9.70
Daily or almost daily	22.17	21.37	20.69	19.96	19.40
Smoking status, %					
Never smoker	57.41	55.91	54.55	53.29	51.41
Former smoker	33.14	34.52	34.97	35.21	34.95
Current smoker <15 cigarettes/day	2.52	2.72	2.93	3.25	3.77
Current smoker, ≥15 cigarettes/day	3.34	3.42	3.90	4.54	5.97
Current smoker, amount unknown	3.32	3.19	3.40	3.47	3.59
With neurotic behaviors (yes/no)					
Mood swings, %	3.36	24.31	51.23	76.11	94.11
Miserableness, %	3.93	23.87	47.95	70.68	90.44
Irritability, %	3.27	14.83	27.43	42.60	69.59
Sensitivity/hurt feelings, %	9.63	45.72	65.88	81.26	94.05
Fed-up feelings, %	2.73	19.84	43.90	67.98	90.14
Nervous feelings, %	0.43	6.87	19.69	32.82	69.79
Worrier/anxious feelings, %	8.49	44.77	67.24	82.81	96.85
Tense/highly strung, %	0.46	3.78	10.60	23.12	62.33
Worry too long after embarrassment, %	6.08	37.77	54.64	69.82	89.85
Suffer from nerves, %	1.66	8.03	16.07	27.55	65.60
Loneliness, isolation, %	1.02	6.40	14.45	26.52	54.60
Guilty feelings, %	1.64	12.87	28.17	44.81	74.42

BMI, body mass index; MET, metabolic equivalent.

a Values are mean (SD).

The association between neuroticism and risk of lung cancer is shown in **Table 2**. Neuroticism was positively associated with lung cancer risk in Model 1 (HR _Q5_ *_vs_*_. Q1_=1.58, 95% CI: 1.35-1.83), although the estimates were substantially attenuated in model additional adjustment for lifestyle factors (smoking status, alcohol intake, BMI and physical activity) (Model 2: HR _Q5 vs. Q1_=1.27, 95% CI: 1.09-1.48). Per 1-SD increment in neuroticism was associated with a 7% higher lung cancer risk (Model 2: HR=1.07; 95% CI: 1.02-1.12). In sensitivity analyses, the associations were basically unchanged after excluding individuals with less than one year of follow-up (**Supplementary Table S3**) and using Fine-Gray models treating the death as a competing risk (**Supplementary Table S4**).

**Table 2:** Association between neuroticism and risk of incident lung cancer.

	No. cases/Person years	Model 1^a^		Model 2^b^	
		HR (95%CI)	*P* value	HR (95%CI)	*P* value
Quintiles					
Q1 (0–1)	386/693001	1.00 (ref)		1.00 (ref)	
Q2 (2–3)	342/566754	1.14 (0.99-1.32)	0.075	1.10 (0.95-1.28)	0.182
Q3 (4–5)	301/489278	1.23 (1.06-1.44)	0.007	1.13 (0.97-1.32)	0.105
Q4 (6–7)	232/377210	1.29 (1.10-1.52)	0.002	1.14 (0.97-1.35)	0.115
Q5 (8–12)	312/450388	1.58 (1.35-1.83)	<0.001	1.27 (1.09-1.48)	0.002
*P* value for trend		<0.001		0.003	
HR per 1-SD increment^c^		1.16 (1.1-1.22)	<0.001	1.07 (1.02-1.12)	0.010

HR, hazards ratio; CI, confidence interval; ref, reference.

a Model 1: adjusted for age at recruitment, sex, ethnic background, education, and family history of lung cancer.

b Model 2: additionally adjusted for smoking status, alcohol intake frequency, BMI and physical activity.

c SD was the standard deviation of neuroticism, which was 3.27.

Similar positive associations were observed in the stratified analyses according to age at recruitment, sex, ethnic background, education, family history of lung cancer, smoking status, alcohol intake frequency, BMI, physical activity and histological subtypes (all *P*_heterogeneity_ > 0.05) (**Supplementary Table S5**). Examination of individual neuroticism items showed that mood swings (HR=1.19, 95% CI: 1.07-1.31), miserableness (HR=1.16, 95%CI: 1.05-1.28), irritability (HR=1.13, 95% CI: 1.01-1.26), and fed up feelings (HR=1.34, 95% CI: 1.21-1.48) were positively associated with lung cancer risk (**Supplementary Table S6**).

**Figure 1:**
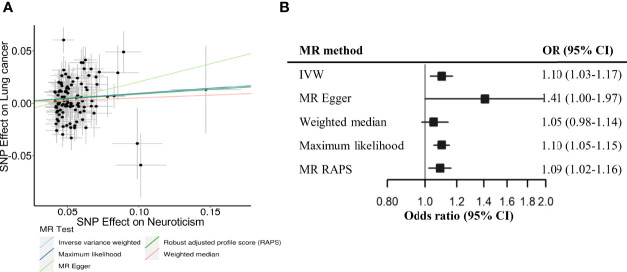
Scatterplot **(A)** and forest plot **(B)** depicting Mendelian randomization (MR) results for neuroticism and risk of lung cancer. N_SNPs_, number of SNP instruments used in the MR analysis; OR, odds ratio; CI, confidence interval; IVW, Inverse variance weighted; RAPS, Robust adjusted profile score - Huber loss function.

In MR analysis, we obtained 107 SNPs for neuroticism after excluding palindromic SNPs or unavailable SNPs in lung cancer GWAS dataset. A genetically predicted neuroticism was also associated with an increased risk of lung cancer (OR _IVW_=1.10, 95% CI: 1.03-1.17), and the association was stable in sensitivity analyses using other MR methods (**Figure 1**). The MR Egger intercept tests yielded no indication of directional horizontal pleiotropy (*P*_intercept_=0.147), but Cochran’s Q test suggested significant heterogeneity among estimates obtained from individual SNPs (*P*=1.69×10^-6^). Furthermore, we identified 9 genetic instruments that were also associated with smoking or BMI in the PhenoScanner database, but similar associations were observed after removing these variants (**Supplementary Table S7**). Additionally, we performed a reverse-direction MR analysis, showing that the genetically instrumented lung cancer was not associated with neuroticism (**Supplementary Table S8**).

**Figure 2:**
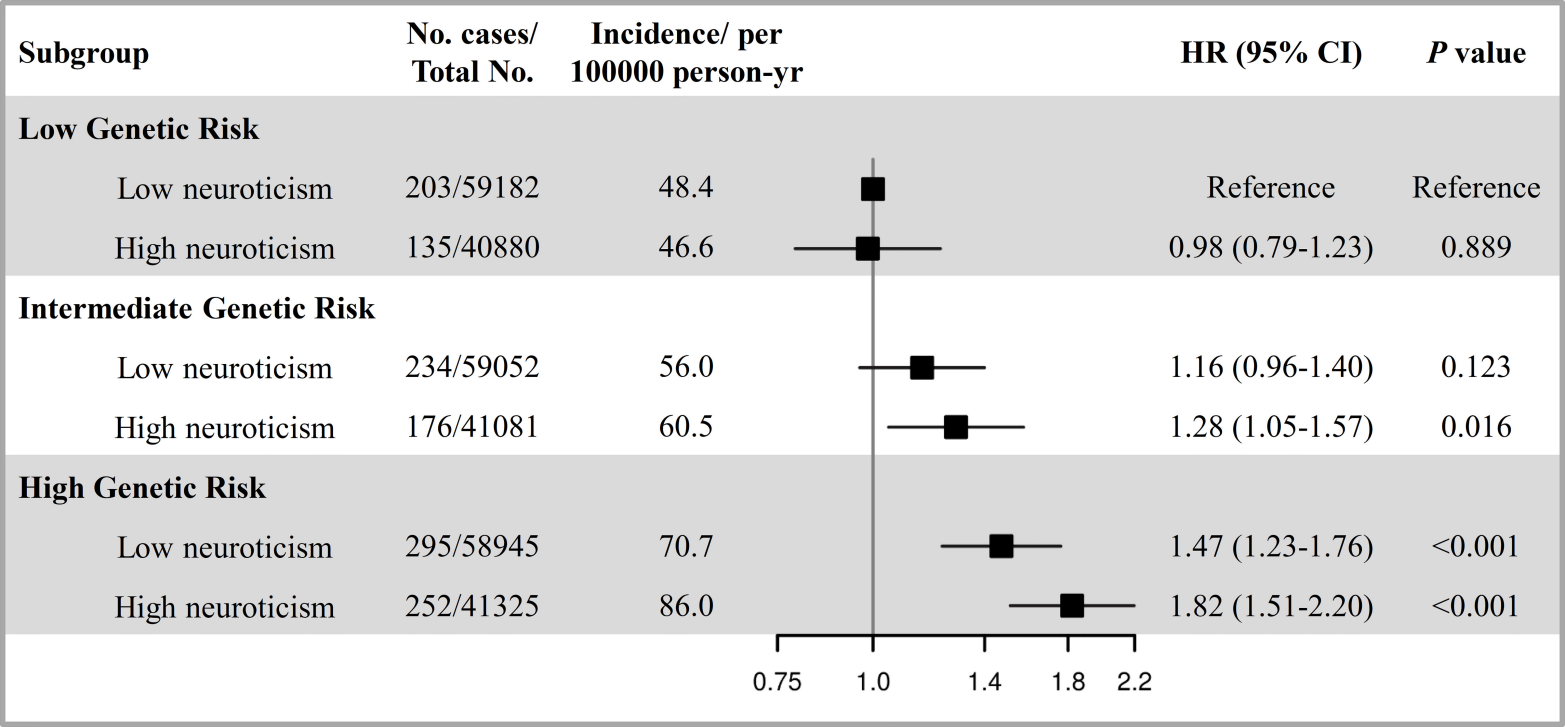
The joint effect and additive interaction of neuroticism and genetic risk with the risk of incident lung cancer. The genetic risk was categorized into low (lowest tertile), intermediate (second tertile), and high (highest tertile). The neuroticism was defined as low and high according to median level. HRs and 95% CIs were estimated using Cox proportional-hazard models with adjustment for age at recruitment, sex, ethnic background, education, family history of lung cancer, smoking status, alcohol intake frequency, BMI, physical activity, the first ten principal components of ancestry and genotyping batch.

The PRS of lung cancer was significantly associated with an increased risk of incident lung cancer (**Supplementary Table S9** and **Supplementary Figures S2A, B**). When combing neuroticism and genetic risk, we observed the joint effect of neuroticism and PRS the risk of incident lung cancer showed a dose-response manner (*P*_trend_ < 0.001, **Figure 2**). Specifically, compared with participants with low PRS and low neuroticism, those with high PRS and high neuroticism had the highest risk of incident lung cancer (HR=1.82, 95%CI: 1.51-2.20). Meanwhile, there was a positive additive interaction between neuroticism and PRS but no multiplicative interaction (**Supplementary Table S10**). In addition, among participants with a high PRS, high neuroticism was associated with a 21% increased risk of lung cancer (HR _High vs. Low_=1.21, 95%CI: 1.02-1.44) than those with low neuroticism (**Supplementary Table S11**), indicating that even in the context of high genetic risk, low neuroticism implied a reduced risk of lung cancer.

## Discussion

In this large-scale prospective study, we observed that higher neuroticism was associated with an increased risk of incident lung cancer. Consistently, the MR analysis demonstrated that a positive causal association of neuroticism with lung cancer. Further, when examining the joint effects of neuroticism and genetic risk on lung cancer risk, we demonstrated that the greatest relative increase of risk was among those with high neuroticism and high genetic risk, and there was a positive additive interaction.

Previous studies of neuroticism and lung cancer have led to inconsistent results, and most of these studies found null or weak positive associations that did not reach the statistical threshold, which may partly be due to study designs and small sample sizes. To our knowledge, this is the largest study to date to examine the association between neuroticism and lung cancer incidence (including 1573 cases), thereby having greater statistical power to detect existing associations. In line with our study, a population-based cohort study comprised 59,548 Swedish and Finnish participants also showed a positive association between neuroticism and lung cancer (18). To exclude the interference of confounding factors, we further carried out MR analyses, which confirmed a causal link between neuroticism and lung cancer. Meanwhile, our MR results were robust to numerous sensitivity analyses for confounding, horizontal pleiotropy, and reverse causality. These findings together suggested that neuroticism may be one etiological factor for lung cancer.

Several underlying mechanisms may mediate the association between neuroticism and lung cancer. Biologically, neuroticism may lead to dysregulation of the immune and endocrine systems (11) and an increase in chronic inflammation (42). Higher neuroticism has been reported to be associated with the atypical response of natural killer cells to stress (43), blunted cortisol response to stress (44) and higher IL6, CRP, and WBC counts (45, 46). Besides, stress may also enhance carcinogenesis through changes in DNA repair and/or apoptosis (47). Moreover, individuals with higher neuroticism tend to live less healthy lifestyles, including cigarette smoking (48), alcohol consumption (49), obesity (50) and physical inactivity (51), which may lead to an increased risk of lung cancer. However, the exact underlying mechanisms linking neuroticism to lung cancer still need to be elucidated by further research.

Given that both genetic and behavioral factors may contribute to disease risk collectively, we assessed the joint effect and interaction between neuroticism and genetic factors on lung cancer. Interestingly, we observed an additive interaction between neuroticism and the genetic risk of lung cancer, which revealed that individuals with high genetic risk and high neuroticism synergistically increased the risk of lung cancer, and this form of interaction indicates that there is a biological interaction between risk factors (52). It suggests that individuals with high genetic risk and high neuroticism should pay more attention to their health. Besides, it may be used to guide screening to identify at-risk persons at an early stage.

The current study is the largest and most comprehensive study to investigate the role of neuroticism in the development of lung cancer, we applied two complementary observational and MR analyses, and examined potential joint effects between neuroticism and genetic susceptibility on lung cancer risk, for informing risk stratification and precision preventive strategies. However, several limitations also need to be acknowledged. First, the UK Biobank participants were also more likely to be more educated and healthier, which may not be generalizable to the general UK population due to “healthy volunteer bias” (53). Second, neuroticism was self-reported data at baseline, and therefore, they may have been misclassified. However, the misclassification was more likely to be non-differential and tended to underestimate the magnitude of association. Finally, the generalizability of genetic analyses is limited to individuals of European descent; therefore, the generalization of the results to other populations should be interpreted with caution.

In conclusion, the current study indicated that neuroticism plays a causal role in the development of lung cancer. Moreover, neuroticism and genetic risk jointly contributed to lung cancer incidence. Further studies are needed to confirm our findings in other populations and elucidate the underlying mechanisms.

## Data Availability Statement

The original contributions presented in the study are included in the article/**Supplementary Material**. Further inquiries can be directed to the corresponding authors.

## Ethics Statement

The ethical approval was obtained from North West Multi-centre Research Ethics Committee (REC reference: 11/NW/03820). The patients/participants provided their written informed consent to participate in this study.

## Author Contributions

The authors’ responsibilities were as follows—XW, XJ, DH, and HM: conceived and designed the research. XW and MZ: performed the statistical analyses. XZ, XF, MJ, YH, and YW offered statistical support during the study. XW and XJ drafted the manuscript. JX, RY, JD, GJ, LX, LD, and ZH: critically revised the manuscript for important intellectual content. All authors contributed to interpretation of the results, reviewed the manuscript for important intellectual content, and read and approved the final manuscript.

## Funding

This work was supported by National Natural Science Foundation of China (81922061, 81803306); Natural Science Foundation of Jiangsu Province (BK20180675); CAMS Innovation Fund for Medical Sciences (2019RU038); and National Science Foundation for Post-doctoral Scientists of China (2018M640466).

## Conflict of Interest

The authors declare that the research was conducted in the absence of any commercial or financial relationships that could be construed as a potential conflict of interest.

## Publisher’s Note

All claims expressed in this article are solely those of the authors and do not necessarily represent those of their affiliated organizations, or those of the publisher, the editors and the reviewers. Any product that may be evaluated in this article, or claim that may be made by its manufacturer, is not guaranteed or endorsed by the publisher.
