# Supplementary material for: Association Between Neuroticism and Risk of Lung Cancer: Results From Observational and Mendelian Randomization Analyses
Source: Front Oncol. 2022 Feb 14;12:836159. doi: 10.3389/fonc.2022.836159 (PMC8882734; doi:10.3389/fonc.2022.836159)
Supplement: Supplementary file 1 [file DataSheet_1.docx]

Supplementary Material

**Title: Association between neuroticism and risk of lung cancer: results from observational and Mendelian randomization analyses**

Xiaoxia Wei^1†^, Xiangxiang Jiang^1†^, Xu Zhang^1^, Xikang Fan^1^, Mengmeng Ji^1^, Yanqian Huang^1^, Jing Xu^2^, Rong Yin^3^, Yuzhuo Wang^1^, Meng Zhu^1,3,4^, Lingbin Du^5,6^, Juncheng Dai^1,4^, Guangfu Jin^1,4^, Lin Xu^3^, Zhibin Hu^1,4^, Dong Hang^1,4*^, Hongxia Ma^1, 4,7*^

**Contents**

**Supplementary Table S1.** Genetic variants used in instrumental variable analysis for neuroticism

**Supplementary Table S2.** Eighteen genome-wide significant (*P* <5×10^-8^) SNPs associated with lung cancer in the study of McKay et al. (2017)

**Supplementary Table S3.** Association between neuroticism and lung cancer risk after excluding participants with less than one year follow-up

**Supplementary Table S4.** Association between neuroticism and lung cancer risk by Competing Risk Analysis

**Supplementary Table S5.** Subgroup analyses of the association between neuroticism and lung cancer risk

**Supplementary Table S6.** Association between neuroticism items and lung cancer risk in the UK Biobank

**Supplementary Table S7.** Association between genetic liability to neuroticism and risk of lung cancer after manual pruning genetic instruments associated with smoking or BMI (*P* <5×10^-8^)

**Supplementary Table S8.** Results of inverse MR analyses

**Supplementary Table S9.** Association between lung cancer polygenic risk score and the risk of incident lung cancer in the UK Biobank

**Supplementary Table S10.** The additive and multiplicative interaction between neuroticism and genetic categories

**Supplementary Table S11.** Association between neuroticism and risk of lung cancer stratified by genetic risk categories

**Supplementary Figure S1.** Study sample flow diagram

**Supplementary Figure S2.** The relationship of polygenic risk score with incident lung cancer in the UK Biobank

| **Supplementary Table S1. Genetic variants used in instrumental variable analysis for neuroticism** | | | | | | | | | | |  |  |
| --- | --- | --- | --- | --- | --- | --- | --- | --- | --- | --- | --- | --- |
| **SNP** | **Effect allele** | **Other allele** | **Neuroticism GWAS** | | |  | **Lung cancer GWAS** | | | | |  |
|  |  |  | **Beta** | **SE** | ***P* value** |  | **Beta** | **SE** | ***P* value** | | |  |
| rs1002655 | G | C | -0.057 | 0.008 | 2.30×10^-12^ |  | 0.002 | 0.013 | 0.904 | | |  |
| rs12127965 | A | G | -0.046 | 0.007 | 6.50×10^-10^ |  | -0.006 | 0.012 | 0.613 | | |  |
| rs17096778 | G | A | -0.099 | 0.018 | 2.40×10^-8^ |  | 0.038 | 0.034 | 0.261 | | |  |
| rs2488401 | T | C | -0.055 | 0.009 | 2.00×10^-9^ |  | 0.021 | 0.014 | 0.142 | | |  |
| rs2883027 | G | A | 0.042 | 0.008 | 1.70×10^-8^ |  | -0.026 | 0.012 | 0.030 | | |  |
| rs10172342 | G | A | 0.047 | 0.009 | 3.20×10^-8^ |  | -0.001 | 0.013 | 0.947 | | |  |
| rs1019706 | G | A | -0.053 | 0.008 | 8.20×10^-11^ |  | 0.003 | 0.013 | 0.821 | | |  |
| rs1109027 | G | A | -0.056 | 0.009 | 3.10×10^-10^ |  | 0.006 | 0.014 | 0.654 | | |  |
| rs1226413 | G | C | 0.062 | 0.009 | 1.50×10^-11^ |  | -0.004 | 0.015 | 0.806 | | |  |
| rs12466146 | C | T | -0.044 | 0.007 | 3.00×10^-9^ |  | -0.001 | 0.012 | 0.963 | | |  |
| rs1431071 | T | G | -0.050 | 0.008 | 6.00×10^-10^ |  | -0.010 | 0.013 | 0.456 | | |  |
| rs17583539 | G | A | 0.050 | 0.009 | 2.10×10^-8^ |  | 0.016 | 0.014 | 0.269 | | |  |
| rs2717043 | T | C | 0.051 | 0.008 | 2.60×10^-11^ |  | 0.014 | 0.012 | 0.255 | | |  |
| rs34668726 | G | C | 0.060 | 0.010 | 1.80×10^-9^ |  | 0.006 | 0.016 | 0.714 | | |  |
| rs4132462 | T | C | 0.045 | 0.008 | 2.30×10^-8^ |  | 0.012 | 0.013 | 0.353 | | |  |
| rs4673866 | G | A | 0.067 | 0.011 | 3.30×10^-10^ |  | 0.002 | 0.017 | 0.892 | | |  |
| rs61038084 | G | A | -0.046 | 0.008 | 2.30×10^-8^ |  | -0.027 | 0.013 | 0.044 | | |  |
| rs72932350 | C | T | 0.056 | 0.010 | 2.40×10^-8^ |  | -0.018 | 0.016 | 0.276 | | |  |
| rs7582403 | G | A | 0.062 | 0.007 | 1.70×10^-16^ |  | 0.033 | 0.012 | 0.005 | | |  |
| rs1542212 | G | T | 0.054 | 0.008 | 1.70×10^-12^ |  | -0.003 | 0.012 | 0.832 | | |  |
| rs2015971 | T | C | -0.042 | 0.007 | 1.30×10^-8^ |  | 0.004 | 0.012 | 0.731 | | |  |
| rs2278609 | C | T | 0.058 | 0.009 | 1.40×10^-10^ |  | 0.000 | 0.014 | 0.998 | | |  |
| rs3772912 | G | A | -0.044 | 0.008 | 1.10×10^-8^ |  | 0.001 | 0.012 | 0.943 | | |  |
| rs3849470 | C | T | -0.044 | 0.007 | 3.40×10^-9^ |  | 0.001 | 0.012 | 0.933 | | |  |
| rs4585149 | C | T | 0.057 | 0.010 | 3.60×10^-9^ |  | 0.007 | 0.015 | 0.632 | | |  |
| rs55970317 | G | T | -0.052 | 0.008 | 3.80×10^-12^ |  | 0.023 | 0.012 | 0.052 | | |  |
| rs836927 | A | C | 0.051 | 0.008 | 8.60×10^-12^ |  | 0.025 | 0.012 | 0.036 | | |  |
| rs931235 | C | A | -0.046 | 0.008 | 7.50×10^-10^ |  | 0.013 | 0.012 | 0.277 | | |  |
| rs9845443 | T | C | 0.047 | 0.008 | 1.00×10^-8^ |  | 0.032 | 0.013 | 0.013 | | |  |
| rs10032297 | T | A | 0.046 | 0.008 | 1.10×10^-9^ |  | 0.016 | 0.012 | 0.168 | | |  |
| rs1372517 | A | G | 0.043 | 0.007 | 4.70×10^-9^ |  | -0.007 | 0.012 | 0.530 | | |  |
| rs7696796 | A | G | 0.049 | 0.009 | 1.30×10^-8^ |  | 0.011 | 0.013 | 0.417 | | |  |
| rs10045508 | G | T | 0.052 | 0.009 | 7.20×10^-9^ |  | -0.019 | 0.014 | 0.173 | | |  |
| rs10476484 | G | A | 0.049 | 0.008 | 3.20×10^-9^ |  | 0.014 | 0.013 | 0.273 | | |  |
| rs35267052^*^ | G | T | 0.078 | 0.012 | 1.60×10^-10^ |  | 0.006 | 0.019 | 0.741 | | |  |
| rs2269426 | A | G | 0.053 | 0.008 | 6.60×10^-12^ |  | -0.033 | 0.012 | 0.006 | | |  |
| rs240788 | G | T | 0.043 | 0.007 | 1.40×10^-8^ |  | -0.008 | 0.012 | 0.482 | | |  |
| rs6916891^*^ | T | G | 0.070 | 0.011 | 9.90×10^-10^ |  | -0.003 | 0.018 | 0.874 | | |  |
| rs872576 | T | C | 0.043 | 0.008 | 4.50×10^-8^ |  | -0.015 | 0.012 | 0.215 | | |  |
| rs9341575 | C | A | 0.046 | 0.008 | 1.10×10^-8^ |  | 0.011 | 0.013 | 0.371 | | |  |
| rs11509880 | A | G | 0.045 | 0.008 | 1.30×10^-8^ |  | 0.020 | 0.012 | 0.108 | | |  |
| rs13226841 | C | T | 0.052 | 0.007 | 1.30×10^-12^ |  | -0.002 | 0.012 | 0.881 | | |  |
| rs13239186^*^ | T | C | 0.047 | 0.008 | 6.60×10^-9^ |  | -0.022 | 0.013 | 0.085 | | |  |
| rs34021161 | T | C | 0.044 | 0.007 | 5.00×10^-9^ |  | 0.000 | 0.012 | 0.981 | | |  |
| rs35627347 | C | T | -0.041 | 0.008 | 4.80×10^-8^ |  | -0.011 | 0.012 | 0.365 | | |  |
| rs4523180 | G | T | -0.069 | 0.013 | 4.50×10^-8^ |  | 0.009 | 0.023 | 0.680 | | |  |
| rs56226325^*^ | T | C | -0.061 | 0.010 | 2.10×10^-9^ |  | -0.039 | 0.016 | 0.012 | | |  |
| rs59970005 | T | C | -0.060 | 0.009 | 5.50×10^-11^ |  | -0.002 | 0.015 | 0.887 | | |  |
| rs1962104 | C | T | 0.041 | 0.008 | 3.50×10^-8^ |  | 0.002 | 0.012 | 0.869 | | |  |
| rs2407746 | G | C | 0.050 | 0.008 | 6.60×10^-10^ |  | -0.003 | 0.013 | 0.840 | | |  |
| rs2721939 | T | C | 0.042 | 0.008 | 2.60×10^-8^ |  | -0.020 | 0.012 | 0.104 | | |  |
| rs2921036^*^ | C | T | -0.083 | 0.007 | 9.20×10^-29^ |  | -0.007 | 0.012 | 0.548 | | |  |
| rs6530964 | T | C | 0.052 | 0.008 | 1.20×10^-10^ |  | -0.003 | 0.014 | 0.817 | | |  |
| rs10119773 | G | A | 0.049 | 0.007 | 4.80×10^-11^ |  | -0.001 | 0.012 | 0.939 | | |  |
| rs10812851 | C | T | -0.048 | 0.008 | 4.80×10^-10^ |  | -0.006 | 0.012 | 0.614 | | |  |
| rs2094580 | T | G | -0.057 | 0.008 | 4.90×10^-12^ |  | -0.024 | 0.013 | 0.067 | | |  |
| rs28427480 | C | A | 0.089 | 0.013 | 2.40×10^-12^ |  | 0.049 | 0.020 | 0.012 | | |  |
| rs56116032 | G | A | -0.063 | 0.009 | 1.90×10^-12^ |  | -0.015 | 0.015 | 0.293 | | |  |
| rs7027172 | A | G | -0.147 | 0.025 | 2.50×10^-9^ |  | -0.013 | 0.042 | 0.754 | | |  |
| rs7869969 | G | A | -0.050 | 0.008 | 2.90×10^-10^ |  | 0.021 | 0.012 | 0.087 | | |  |
| rs9298995 | A | G | -0.045 | 0.008 | 2.40×10^-9^ |  | 0.006 | 0.012 | 0.597 | | |  |
| rs2791459 | A | C | -0.047 | 0.008 | 4.70×10^-10^ |  | 0.009 | 0.012 | 0.467 | | |  |
| rs703409 | T | C | -0.050 | 0.009 | 4.20×10^-9^ |  | -0.001 | 0.013 | 0.958 | | |  |
| rs7093157 | T | G | -0.044 | 0.008 | 4.60×10^-8^ |  | 0.011 | 0.012 | 0.385 | | |  |
| rs11603808^*^ | T | C | -0.053 | 0.007 | 2.00×10^-12^ |  | 0.000 | 0.012 | 0.995 | | |  |
| rs167915 | T | A | -0.048 | 0.008 | 7.10×10^-10^ |  | -0.002 | 0.012 | 0.844 | | |  |
| rs1903844 | C | T | -0.049 | 0.008 | 3.40×10^-10^ |  | -0.028 | 0.012 | 0.023 | | |  |
| rs1940735^*^ | G | T | 0.056 | 0.009 | 4.80×10^-11^ |  | 0.005 | 0.013 | 0.699 | | |  |
| rs2071754 | T | C | -0.067 | 0.009 | 4.80×10^-13^ |  | -0.033 | 0.015 | 0.024 | | |  |
| rs297343 | G | T | -0.050 | 0.008 | 1.10×10^-10^ |  | -0.003 | 0.012 | 0.826 | | |  |
| rs674094 | C | A | -0.060 | 0.008 | 1.70×10^-13^ |  | -0.039 | 0.013 | 0.002 | | |  |
| rs7107356^*^ | G | A | 0.053 | 0.007 | 9.30×10^-13^ |  | 0.018 | 0.012 | 0.128 | | |  |
| rs7111031 | A | C | 0.070 | 0.008 | 6.80×10^-20^ |  | 0.025 | 0.012 | 0.039 | | |  |
| rs10745624 | C | T | 0.044 | 0.007 | 5.30×10^-9^ |  | -0.017 | 0.012 | 0.161 | | |  |
| rs2280711 | T | C | 0.063 | 0.010 | 6.30×10^-10^ |  | -0.023 | 0.016 | 0.138 | | |  |
| rs4267163 | A | G | 0.043 | 0.008 | 4.60×10^-8^ |  | -0.007 | 0.013 | 0.562 | | |  |
| rs6606710 | C | T | 0.062 | 0.008 | 2.70×10^-15^ |  | -0.007 | 0.013 | 0.571 | | |  |
| rs877995 | A | G | 0.061 | 0.009 | 9.70×10^-11^ |  | -0.015 | 0.015 | 0.287 | | |  |
| rs1892350 | G | A | 0.044 | 0.007 | 2.30×10^-9^ |  | 0.011 | 0.012 | 0.348 | | |  |
| rs9516861 | A | T | 0.064 | 0.011 | 1.10×10^-8^ |  | 0.013 | 0.017 | 0.461 | | |  |
| rs9517313 | C | G | 0.043 | 0.008 | 1.10×10^-8^ |  | 0.008 | 0.012 | 0.523 | | |  |
| rs11627348 | A | C | 0.065 | 0.010 | 4.20×10^-10^ |  | 0.027 | 0.017 | 0.111 | | |  |
| rs1782170 | C | A | 0.049 | 0.008 | 3.90×10^-9^ |  | 0.012 | 0.014 | 0.392 | | |  |
| rs35641442 | A | G | 0.060 | 0.007 | 7.60×10^-16^ |  | 0.019 | 0.012 | 0.112 | | |  |
| rs36006259 | T | C | -0.058 | 0.010 | 2.00×10^-8^ |  | 0.003 | 0.017 | 0.856 | | |  |
| rs4140799 | A | G | -0.046 | 0.007 | 8.00×10^-10^ |  | -0.020 | 0.012 | 0.091 | | |  |
| rs4899292 | G | A | 0.049 | 0.008 | 2.90×10^-10^ |  | -0.003 | 0.012 | 0.818 | | |  |
| rs11633354 | C | T | -0.046 | 0.007 | 6.20×10^-10^ |  | -0.019 | 0.012 | 0.113 | | |  |
| rs1563245 | G | T | 0.047 | 0.008 | 4.30×10^-10^ |  | 0.060 | 0.012 | 4.41×10^-7^ | | |  |
| rs57506932 | T | C | -0.067 | 0.011 | 3.10×10^-9^ |  | 0.003 | 0.019 | 0.887 | | |  |
| rs10852673 | A | G | -0.046 | 0.008 | 3.00×10^-8^ |  | 0.002 | 0.013 | 0.881 | | |  |
| rs113199002^†^ | C | G | -0.042 | 0.008 | 3.20×10^-8^ |  | -0.011 | 0.012 | 0.378 | | |  |
| rs2042395 | A | G | -0.054 | 0.009 | 9.40×10^-10^ |  | -0.021 | 0.014 | 0.128 | | |  |
| rs2199036^*^ | T | C | -0.047 | 0.008 | 1.00×10^-9^ |  | 0.030 | 0.012 | 0.016 | | |  |
| rs3785232 | T | C | 0.055 | 0.008 | 4.60×10^-12^ |  | -0.023 | 0.012 | 0.061 | | |  |
| rs7193453 | T | C | -0.042 | 0.007 | 2.00×10^-8^ |  | -0.010 | 0.012 | 0.377 | | |  |
| rs12601333 | G | C | -0.042 | 0.008 | 4.80×10^-8^ |  | 0.003 | 0.012 | 0.813 | | |  |
| rs12938775 | A | G | -0.049 | 0.007 | 3.10×10^-11^ |  | -0.011 | 0.012 | 0.363 | | |  |
| rs6503091 | A | G | 0.044 | 0.008 | 6.60×10^-9^ |  | 0.020 | 0.012 | 0.091 | | |  |
| rs7502590 | G | A | -0.072 | 0.010 | 3.40×10^-12^ |  | -0.030 | 0.017 | 0.074 | | |  |
| rs9908167 | A | G | -0.042 | 0.008 | 4.20×10^-8^ |  | 0.004 | 0.012 | 0.759 | | |  |
| rs11665070 | A | G | -0.071 | 0.008 | 2.70×10^-19^ |  | -0.018 | 0.012 | 0.144 | | |  |
| rs4534926^†^ | C | G | 0.047 | 0.007 | 2.90×10^-10^ |  | 0.019 | 0.012 | 0.114 | | |  |
| rs7235757 | A | G | 0.063 | 0.008 | 6.60×10^-15^ |  | 0.041 | 0.013 | 0.001 | | |  |
| rs77867423 | G | C | -0.085 | 0.015 | 1.30×10^-8^ |  | -0.029 | 0.026 | 0.260 | | |  |
| rs117298864 | A | G | 0.101 | 0.018 | 1.90×10^-8^ |  | -0.059 | 0.030 | 0.053 | | |  |
| rs4578918 | C | T | -0.051 | 0.008 | 1.70×10^-9^ |  | -0.025 | 0.013 | 0.056 | | |  |
| rs910187 | A | G | -0.043 | 0.008 | 1.40×10^-8^ |  | -0.001 | 0.012 | 0.936 | | |  |
| rs11090045 | A | G | 0.059 | 0.008 | 4.00×10^-13^ |  | -0.009 | 0.013 | 0.522 | | |  |
| rs6545135 | T | C | -0.043 | 0.008 | 4.70×10^-8^ |  |  |  |  | | |  |
| rs12203592 | T | C | 0.053 | 0.009 | 1.90×10^-9^ |  |  |  |  | | |  |
| rs10456089 | A | G | -0.084 | 0.014 | 3.80×10^-9^ |  |  |  |  | | |  |
| rs12530421 | C | A | 0.088 | 0.015 | 3.20×10^-9^ |  |  |  |  | | |  |
| rs77335224 | T | C | -0.074 | 0.013 | 1.70×10^-8^ |  |  |  |  | | |  |
| rs753044 | T | C | -0.041 | 0.008 | 4.90×10^-8^ |  |  |  |  | | |  |
| rs17652520 | A | G | 0.095 | 0.009 | 3.10×10^-27^ |  |  |  |  | | |  |
| SNP, single nucleotide polymorphism; CHR, chromosome; EAF, effect allele frequency; SE, standard error | | | | | | | | | | |  |  |
| ^†^Palindromic SNPs with minor allele frequency of > 0.42 were considered to be strand-ambiguous and removed from final analysis. | | | | | | | | | | |  |  |
| ^*^Instruments associated with smoking status or BMI (*P* < 5×10^-8^) were removed from sensitivity analysis | | | | | | | | | |  | | |

| **Supplementary Table S2. Eighteen genome-wide significant (*P* <5×10^-8^) SNPs associated with lung cancer in the study of McKay et al. (2017)** | | | | | | | | | | | | |  |
| --- | --- | --- | --- | --- | --- | --- | --- | --- | --- | --- | --- | --- | --- |
| **SNP** | **CHR** | **Position (hg19/b37)** | **Effect allele** | **other allele** | **EAF** | **BETA** | | **SE** | | ***P* value** | | |  |
| rs71658797 | 1 | 77967507 | A | T | 0.103 | 0.128 | | 0.019 | | 3.25×10^-11^ | | |  |
| rs13080835 | 3 | 189357199 | T | G | 0.493 | -0.111 | | 0.016 | | 7.45×10^-12^ | | |  |
| rs7705526 | 5 | 1285974 | A | C | 0.342 | 0.222 | | 0.018 | | 3.80×10^-35^ | | |  |
| rs6920364 | 6 | 167377165 | C | G | 0.456 | 0.068 | | 0.012 | | 1.29×10^-8^ | | |  |
| rs116822326 | 6 | 31434111 | G | A | 0.155 | 0.223 | | 0.025 | | 3.83×10^-19^ | | |  |
| rs11780471 | 8 | 27344719 | A | G | 0.060 | -0.141 | | 0.025 | | 1.69×10^-8^ | | |  |
| rs4236709 | 8 | 32410110 | G | A | 0.218 | 0.124 | | 0.019 | | 1.28×10^-10^ | | |  |
| rs885518 | 9 | 21830157 | G | A | 0.101 | 0.155 | | 0.025 | | 9.96×10^-10^ | | |  |
| rs11591710 | 10 | 105687632 | C | A | 0.137 | 0.151 | | 0.023 | | 6.30×10^-11^ | | |  |
| rs1056562 | 11 | 118125625 | T | C | 0.473 | 0.102 | | 0.016 | | 2.76×10^-10^ | | |  |
| rs7953330 | 12 | 998819 | C | G | 0.315 | -0.146 | | 0.020 | | 7.26×10^-13^ | | |  |
| rs11571833 | 13 | 32972626 | T | A | 0.011 | 0.472 | | 0.058 | | 6.12×10^-16^ | | |  |
| rs55781567 | 15 | 78857986 | G | C | 0.367 | 0.260 | | 0.012 | | 3.08×10^-103^ | | |  |
| rs66759488 | 15 | 47577451 | A | G | 0.362 | 0.068 | | 0.012 | | 2.83×10^-8^ | | |  |
| rs77468143 | 15 | 49376624 | G | T | 0.253 | -0.155 | | 0.019 | | 1.69×10^-16^ | | |  |
| rs56113850 | 19 | 41353107 | T | C | 0.440 | -0.123 | | 0.014 | | 5.02×10^-19^ | | |  |
| rs41309931 | 20 | 62326579 | T | G | 0.117 | 0.157 | | 0.026 | | 1.31×10^-9^ | | |  |
| rs17879961^‡^ | 22 | 29121087 | G | A | 0.005 | -0.892 | 0.124 | | 5.70×10^-13^ | | |  |  |
| SNP, single nucleotide polymorphism; CHR, chromosome; EAF, effect allele frequency; SE, standard error | | | | | | | | | | |  |  |  |

| **Supplementary Table S3. Association between neuroticism and lung cancer risk after excluding participants with less than one year follow-up** | | | | | | |  | | |  |  |  |  |
| --- | --- | --- | --- | --- | --- | --- | --- | --- | --- | --- | --- | --- | --- |
|  | **No. cases / Person years** | **Model 1 ^a^** | |  | **Model 2 ^b^** | | |  |  |  |  |  |  |
|  |  | **HR (95%CI)** | ***P* value** |  | **HR (95%CI)** | ***P* value** | |  |  |  |  |  |  |
| Quintiles |  |  |  |  |  |  | |  |  |  |  |  |  |
| Q1 (0–1) | 347/692902 | 1.00 (ref) |  |  | 1.00 (ref) |  | |  |  |  |  |  |  |
| Q2 (2–3) | 307/566677 | 1.14 (0.98-1.33) | 0.094 |  | 1.11 (0.95-1.29) | 0.202 | |  |  |  |  |  |  |
| Q3 (4–5) | 273/489207 | 1.25 (1.06-1.46) | 0.007 |  | 1.15 (0.98-1.35) | 0.093 | |  |  |  |  |  |  |
| Q4 (6–7) | 219/377165 | 1.36 (1.15-1.61) | <0.001 |  | 1.20 (1.01-1.43) | 0.035 | |  |  |  |  |  |  |
| Q5 (8–12) | 290/450324 | 1.63 (1.40-1.92) | <0.001 |  | 1.31 (1.12-1.54) | 0.001 | |  |  |  |  |  |  |
| *P* value for trend |  | <0.001 |  |  | 0.001 |  | |  |  |  |  |  |  |
| HR per 1-SD increment **^c^** |  | 1.18 (1.12-1.24) | <0.001 |  | 1.08 (1.03-1.14) | 0.003 | |  |  |  |  |  |  |
| HR, hazards ratio; CI, confidence interval  ^a^ Model 1: adjusted for age at recruitment, sex, ethnic background, education, and family history of lung cancer.  ^b^ Model 2: model1 additionally adjusted for smoking status, alcohol intake frequency, BMI and physical activity  ^c^ SD was the standard deviation of neuroticism, which was 3.27. | | | | | | | | |  |  |  |  |  |

| **Supplementary Table S4. Association between neuroticism and lung cancer risk by Competing Risk Analysis** | | | | | | | | |  |  | |  |  | | |  | |  |
| --- | --- | --- | --- | --- | --- | --- | --- | --- | --- | --- | --- | --- | --- | --- | --- | --- | --- | --- |
|  | **Model 1 ^a^** | |  | **Model 2 ^b^** | | | |  |  |  |  |  |  |  |  |  |  |  |
|  | **HR (95%CI)** | ***P* value** |  | **HR (95%CI)** | | ***P* value** | |  |  |  |  |  |  |  |  |  |  |  |
| Quintiles |  |  |  |  | |  | |  |  |  |  |  |  |  |  |  |  |  |
| Q1 (0–1) | 1.00 (ref) |  |  | 1.00 (ref) | |  | |  |  |  |  |  |  |  |  |  |  |  |
| Q2 (2–3) | 1.14 (0.99-1.32) | 0.077 |  | 1.10 (0.95-1.28) | | 0.190 | |  |  |  |  |  |  |  |  |  |  |  |
| Q3 (4–5) | 1.23 (1.06-1.44) | 0.007 |  | 1.13 (0.97-1.32) | | 0.110 | |  |  |  |  |  |  |  |  |  |  |  |
| Q4 (6–7) | 1.29 (1.09-1.52) | 0.003 |  | 1.14 (0.96-1.34) | | 0.130 | |  |  |  |  |  |  |  |  |  |  |  |
| Q5 (8–12) | 1.57 (1.35-1.82) | <0.001 |  | 1.26 (1.08-1.47) | | 0.003 | |  |  |  |  |  |  |  |  |  |  |  |
| *P* value for trend | <0.001 |  |  | 0.004 | |  | |  |  |  |  |  |  |  |  |  |  |  |
| HR per 1-SD increment **^c^** | 1.16 (1.10-1.22) | <0.001 |  | 1.07 (1.01-1.12) | | 0.011 | |  |  |  |  |  |  |  |  |  |  |  |
| HR, hazards ratio; CI, confidence interval | | | | |  |  |  |  |  |  |  |  |  |  |  |  |  |  |
| ^a^ Model 1: adjusted for age at recruitment, sex, ethnic background, education and family history of lung cancer. | | | | | | |  |  |  |  |  |  |  |  |  |  |  |  |
| ^b^ Model 2: model1 additionally adjusted for smoking status, alcohol intake frequency, BMI and physical activity. | | | | | | |  |  |  |  |  |  |  |  |  |  |  |  |
| ^c^ SD was the standard deviation of neuroticism scores, which was 3.27. | | | | | | | | | | |  | | |  |  | |  | |

| **Supplementary Table S5. Subgroup analyses of the association between neuroticism and lung cancer risk** | | | | | | | | | | |  |  |  |
| --- | --- | --- | --- | --- | --- | --- | --- | --- | --- | --- | --- | --- | --- |
| **Subgroup** | | **No. cases/**  **Total no.** | | **HR per 1-SD increment ^a^** | | | ***P* value** | | ***P* _heterogeneity_** | |  |  |  |
| **Age (years)** | |  | |  | | |  | | 0.061 | |  |  |  |
| <60 | | 437/215147 | | 0.96 (0.88-1.06) | | | 0.449 | |  | |  |  |  |
| ≥60 | | 1136/149304 | | 1.07 (1.01-1.13) | | | 0.018 | |  | |  |  |  |
| **Sex** | |  | |  | | |  | | 0.113 | |  |  |  |
| Women | | 683/191615 | | 1.12 (1.04-1.20) | | | 0.003 | |  | |  |  |  |
| Men | | 890/172836 | | 1.03 (0.97-1.10) | | | 0.350 | |  | |  |  |  |
| **Ethnic background ^b^** | |  | |  | | |  | | 0.389 | |  |  |  |
| White race | | 1532/346544 | | 1.07 (1.02-1.13) | | | 0.006 | |  | |  |  |  |
| Non-white | | 38/16919 | | 0.92 (0.66-1.29) | | | 0.645 | |  | |  |  |  |
| **Education ^b^** | |  | |  | | |  | | 0.338 | |  |  |  |
| College or university degree | | 260/122316 | | 1.01 (0.89-1.14) | | | 0.897 | |  | |  |  |  |
| Non-degree | | 1271/236714 | | 1.08 (1.02-1.14) | | | 0.009 | |  | |  |  |  |
| **Family history of lung cancer** | |  | |  | | |  | | 0.327 | |  |  |  |
| No | | 1243/319678 | | 1.08 (1.02-1.14) | | | 0.006 | |  | |  |  |  |
| Yes | | 330/44773 | | 1.02 (0.91-1.13) | | | 0.748 | |  | |  |  |  |
| **Smoking status ^b^** | |  | |  | | |  | | 0.177 | |  |  |  |
| Never | | 213/200030 | | 1.17 (1.02-1.34) | | | 0.024 | |  | |  |  |  |
| Previous | | 701/125404 | | 1.11 (1.03-1.19) | | | 0.008 | |  | |  |  |  |
| Current | | 652/38059 | | 1.02 (0.94-1.11) | | | 0.592 | |  | |  |  |  |
| **Alcohol intake frequency ^b^** | |  | |  | | |  | | 0.742 | |  |  |  |
| Never/rare | | 707/162139 | | 1.05 (0.97-1.13) | | | 0.215 | |  | |  |  |  |
| Twice or less per week | | 484/135079 | | 1.07 (0.98-1.17) | | | 0.156 | |  | |  |  |  |
| At least three times per week | | 380/67062 | | 1.10 (1.00-1.22) | | | 0.061 | |  | |  |  |  |
| **BMI (kg/m^2^)** | |  | |  | | |  | | 0.839 | |  |  |  |
| <25 | | 491/118978 | | 1.07 (0.98-1.16) | | | 0.154 | |  | |  |  |  |
| 25-30 | | 669/156951 | | 1.06 (0.98-1.14) | | | 0.162 | |  | |  |  |  |
| ≥30 | | 413/88522 | | 1.10 (0.99-1.21) | | | 0.070 | |  | |  |  |  |
| **Physical activity** | |  | |  | | |  | | 0.296 | |  |  |  |
| Low | | 301/56098 | | 1.14 (1.02-1.28) | | | 0.020 | |  | |  |  |  |
| Moderate | | 901/213869 | | 1.07 (1.00-1.14) | | | 0.053 | |  | |  |  |  |
| High | | 371/94484 | | 1.01 (0.91-1.12) | | | 0.814 | |  | |  |  |  |
| **Histological subtypes** | |  | |  | | |  | | 0.651 | |  |  |  |
| Adenocarcinoma | | 644/363522 | | 1.08 (1.00-1.17) | | | 0.042 | |  | |  |  |  |
| Squamous cell carcinoma | | 318/363196 | | 1.05 (0.94-1.17) | | | 0.423 | |  | |  |  |  |
| Small cell carcinoma | | 156/363034 | | 1.00 (0.85-1.17) | | | 0.989 | |  | |  |  |  |
| ^a^ HRs and 95% CIs were estimated using Cox proportional-hazard models with adjustment for age at recruitment, sex, ethnic background, education, family history of lung cancer, smoking status, alcohol intake frequency, BMI and physical activity. SD was the standard deviation of neuroticism, which was 3.27.  ^b^ Missing values were present in the dataset. | | | | | | | | | | |  |  |  |
| **Supplementary Table S6. Association between neuroticism items and lung cancer risk in the UK Biobank** | | | | | | | | | | | |  |  |
| **Items** | **No. cases/**  **Total no.** | | **Model 1 ^a^** | | |  | | **Model 2 ^b^** | | | | | |
|  |  |  | **HR (95% CI)** | | ***P* value** |  | | **HR (95% CI)** | | ***P* value** | | | |
| **Mood swings** |  | |  | |  |  | |  | |  | | | |
| No | 807/205708 | | 1.00 (ref) | |  |  | | 1.00 (ref) | |  | | | |
| Yes | 766/158743 | | 1.39 (1.26-1.54) | | <0.001 |  | | 1.19 (1.07-1.31) | | 0.001 | | | |
| **Miserableness** |  | |  | |  |  | |  | |  | | | |
| No | 865/213007 | | 1.00 (ref) | |  |  | | 1.00 (ref) | |  | | | |
| Yes | 708/151444 | | 1.38 (1.24-1.52) | | <0.001 |  | | 1.16 (1.05-1.28) | | 0.005 | | | |
| **Irritability** |  | |  | |  |  | |  | |  | | | |
| No | 1096/263341 | | 1.00 (ref) | |  |  | | 1.00 (ref) | |  | | | |
| Yes | 477/101110 | | 1.27 (1.14-1.41) | | <0.001 |  | | 1.13 (1.01-1.26) | | 0.027 | | | |
| **Sensitivity/hurt feelings** |  | |  | |  |  | |  | |  | | | |
| No | 758/169568 | | 1.00 (ref) | |  |  | | 1.00 (ref) | |  | | | |
| Yes | 815/194883 | | 1.00 (0.90-1.11) | | 0.994 |  | | 0.96 (0.87-1.06) | | 0.448 | | | |
| **Fed up feelings** |  | |  | |  |  | |  | |  | | | |
| No | 813/221845 | | 1.00 (ref) | |  |  | | 1.00 (ref) | |  | | | |
| Yes | 760/142606 | | 1.67 (1.52-1.85) | | <0.001 |  | | 1.34 (1.21-1.48) | | <0.001 | | | |
| **Nervous feelings** |  | |  | |  |  | |  | |  | | | |
| No | 1205/282939 | | 1.00 (ref) | |  |  | | 1.00 (ref) | |  | | | |
| Yes | 368/81512 | | 1.12 (0.99-1.26) | | 0.066 |  | | 1.08 (0.96-1.21) | | 0.230 | | | |
| **Worrier/anxious feelings** |  | |  | |  |  | |  | |  | | | |
| No | 731/167908 | | 1.00 (ref) | |  |  | | 1.00 (ref) | |  | | | |
| Yes | 842/196543 | | 1.05 (0.95-1.16) | | 0.352 |  | | 1.04 (0.94-1.15) | | 0.425 | | | |
| **Tense/highly strung** |  | |  | |  |  | |  | |  | | | |
| No | 1268/301597 | | 1.00 (ref) | |  |  | | 1.00 (ref) | |  | | | |
| Yes | 305/62854 | | 1.25 (1.10-1.42) | | <0.001 |  | | 1.10 (0.97-1.25) | | 0.139 | | | |
| **Worry after embarrassment** |  | |  | |  |  | |  | |  | | | |
| No | 902/195970 | | 1.00 (ref) | |  |  | | 1.00 (ref) | |  | | | |
| Yes | 671/168481 | | 0.97 (0.88-1.08) | | 0.602 |  | | 1.00 (0.90-1.10) | | 0.946 | | | |
| **Suffer from nerves** |  | |  | |  |  | |  | |  | | | |
| No | 1220/288786 | | 1.00 (ref) | |  |  | | 1.00 (ref) | |  | | | |
| Yes | 353/75665 | | 1.20 (1.06-1.35) | | 0.003 |  | | 1.08 (0.96-1.22) | | 0.196 | | | |
| **Loneliness/ isolation** |  | |  | |  |  | |  | |  | | | |
| No | 1245/299403 | | 1.00 (ref) | |  |  | | 1.00 (ref) | |  | | | |
| Yes | 328/65048 | | 1.37 (1.21-1.55) | | <0.001 |  | | 1.04 (0.91-1.17) | | 0.579 | | | |
| **Guilty feelings** |  | |  | |  |  | |  | |  | | | |
| No | 1153/261733 | | 1.00 (ref) | |  |  | | 1.00 (ref) | |  | | | |
| Yes | 420/ 102718 | | 1.04 (0.93-1.17) | | 0.466 |  | | 0.97 (0.87-1.09) | | 0.658 | | | |
| ^a^ Model 1: adjusted for age at recruitment, sex, ethnic background, education, and family history of lung cancer. | | | | | | | | | | | | |  |
| ^b^ Model 2: model1 additionally adjusted for smoking status, alcohol intake frequency, BMI and physical activity | | | | | | | | | | | | |  |

| **Supplementary Table S7. Association between genetic liability to neuroticism and risk of lung cancer after manual pruning genetic instruments associated with smoking or BMI (*P* <5×10^-8^)** | | | | |  |  |  |
| --- | --- | --- | --- | --- | --- | --- | --- |
| **Outcome** | **N_SNPs_** | **MR Estimator** | **OR (95% CI)** | ***P* value** | |  |  |
| Lung cancer (cases: n=29,266, controls: n=56,450) | 98 | IVW | 1.10 (1.03-1.18) | 0.004 | |  |  |
|  |  | MR Egger | 1.41 (0.97-2.05) | 0.077 | |  |  |
|  |  | Weighted median | 1.04 (0.96-1.13) | 0.339 | |  |  |
|  |  | Maximum likelihood | 1.10 (1.05-1.16) | <0.001 | |  |  |
|  |  | MR RAPS | 1.09 (1.02-1.17) | 0.011 | |  |  |
| N_SNPs_, number of SNP instruments used in the MR analysis; OR, odds ratio; CI, confidence interval; IVW, Inverse variance weighted; RAPS, Robust adjusted profile score - Huber loss function | | | | | | |  |
|  | | | | | | | |

| **Supplementary Table S8. Results of** **inverse MR analyses** | | | | | |  |
| --- | --- | --- | --- | --- | --- | --- |
| **Outcome** | **N_SNPs_** | **MR method** | **Beta** | **SE** | ***P* value** |  |
| Neuroticism score (374,323 individuals) | 17 | IVW | -0.013 | 0.029 | 0.659 |  |
|  |  | MR Egger | -0.054 | 0.080 | 0.509 |  |
|  |  | Weighted median | -0.002 | 0.024 | 0.935 |  |
|  |  | Maximum likelihood | -0.013 | 0.015 | 0.375 |  |
|  |  | MR RAPS | -0.019 | 0.029 | 0.519 |  |
| N_SNPs_, number of SNP instruments used in the MR analysis; Beta: estimate of causal effect of exposure on the outcome; SE: standard error of beta; IVW, Inverse variance weighted; RAPS, Robust adjusted profile score - Huber loss function  **^†^**rs6920364 was palindromic SNPs with minor allele frequency of >0.42 were considered to be strand-ambiguous and removed from final analysis, and rs17879961 was not available in the neuroticism score GWAS and the closely related SNP’s (r^2^ = 1) rs185936232 were used instead | | | | | | |

| **Supplementary Table S9. Association between lung cancer polygenic risk score and the risk of incident lung cancer in the UK Biobank** | | | | | | |  |
| --- | --- | --- | --- | --- | --- | --- | --- |
| **Genetic risk** | **No. cases / person years** | **Model 1 ^a^** | |  | **Model 2 ^b^** | | |
|  |  | **HR (95%CI)** | ***P* value** |  | **HR (95%CI)** | ***P* value** | |
| Tertiles |  |  |  |  |  |  | |
| Low | 338/708592 | 1.00 (ref) |  |  | 1.00 (ref) |  | |
| Intermediate | 410/708948 | 1.22 (1.05-1.4) | 0.008 |  | 1.22 (1.05-1.41) | 0.007 | |
| High | 547/709918 | 1.61 (1.40-1.84) | <0.001 |  | 1.62 (1.42-1.86) | <0.001 | |
| *P* value for trend |  | <0.001 |  |  | <0.001 |  | |
| HR per 1-SD increment ^c^ |  | 1.22 (1.16-1.29) | <0.001 |  | 1.23 (1.16-1.30) | <0.001 | |
| HR, hazards ratio; CI, confidence interval | | | | | | |  |
| ^a^ Model 1: adjusted for age at recruitment, sex, the first ten principal components of ancestry and genotyping batch. | | | | | | | |
| ^b^ Model 2: model1 additionally adjusted for education, family history of lung cancer, smoking status, alcohol intake frequency, BMI and physical activity. | | | | | | | |
| ^c^ SD was the standard deviation of lung cancer polygenic risk score, which was 0.37. | | | | | | | |

| **Supplementary Table S10. The additive and multiplicative interaction between neuroticism and genetic categories ^a^** | | | | |
| --- | --- | --- | --- | --- |
| **Genetic risk** | **Additive interaction ^b^** | |  | **Multiplicative interaction** |
|  | **RERI (95%CI)** | **AP (95%CI)** |  | ***P* interaction** |
| Intermediate | 0.14 (-0.18-0.46) | 0.11 (-0.13-0.35) |  | 0.173 |
| High | 0.37 (0.03-0.70) | 0.20 (0.02-0.38) |  |  |
| RERI, relative excess risk due to interaction; AP, attributable proportion due to interaction; CI, confidence interval | | | | |
| ^a^ Adjusted for age at recruitment, sex, ethnic background, education, family history of lung cancer, smoking status, alcohol intake frequency, BMI, physical activity, the first ten principal components of ancestry and genotyping batch. | | | | |
| ^b^ To estimate RERI and AP, the low neuroticism and the lowest genetic risk (low PRS) groups were the reference categories. | | | | |

| **Supplementary Table S11. Association between neuroticism and risk of lung cancer stratified by genetic risk categories** | | | | | | | |  |  |
| --- | --- | --- | --- | --- | --- | --- | --- | --- | --- |
| **Subgroup** | **No. cases / Person years** | **Model 1 ^a^** | |  | **Model 2 ^b^** | | |  |  |
|  |  | **HR (95%CI)** | ***P* value** |  | **HR (95%CI)** | ***P* value** | |  |  |
| **Low Genetic Risk** |  |  |  |  |  |  | |  |  |
| Low neuroticism | 203/419060 | 1.00 (ref) |  |  | 1.00 (ref) |  | |  |  |
| High neuroticism | 135/289533 | 1.08 (0.87-1.35) | 0.482 |  | 0.97 (0.78-1.21) | 0.795 | |  |  |
| **Intermediate Genetic Risk** |  |  |  |  |  |  | |  |  |
| Low neuroticism | 234/418008 | 1.00 (ref) |  |  | 1.00 (ref) |  | |  |  |
| High neuroticism | 176/290940 | 1.20 (0.98-1.46) | 0.075 |  | 1.09 (0.89-1.33) | 0.403 | |  |  |
| **High Genetic Risk** |  |  |  |  |  |  | |  |  |
| Low neuroticism | 295/417005 | 1.00 (ref) |  |  | 1.00 (ref) |  | |  |  |
| High neuroticism | 252/292913 | 1.34 (1.13-1.59) | 0.001 |  | 1.21 (1.02-1.44) | 0.031 | |  |  |
| HR, hazard ratio; CI, confidence interval; Low genetic risk: lowest tertile of lung cancer PRS; Intermediate genetic risk: second tertile of lung cancer PRS; High genetic risk: highest tertile of lung cancer PRS; Low neuroticism: ≤ median neuroticism score; High neuroticism: > median neuroticism score | | | | | | | | |  |
| ^a^ Model 1: adjusted for age at recruitment, sex, the first ten principal components of ancestry and genotyping batch. | | | | | | | | | |
| ^b^ Model 2: model1 additionally adjusted for education, family history of lung cancer, smoking status, alcohol intake frequency, BMI and physical activity. | | | | | | | | |  |

**Figure Legends**

**Supplementary Figure S1. Study sample flow diagram**

**Supplementary Figure S2.** **The relationship of polygenic risk score with incident lung cancer in the UK Biobank.** (A) Linear relationship between polygenic risk score (PRS) and lung cancer risk was assessed using restricted cubic spline analysis; (B) Individuals in the UK Biobank were divided into five groups according to their PRS, and HRs for each group were compared with those in quintile 1 (reference group) of PRS; error bars show the 95% CIs.

HRs and 95% CIs were estimated using Cox proportional-hazard models with adjustment for age at recruitment, sex, ethnic background, education, family history of lung cancer, smoking status, alcohol intake frequency, BMI, physical activity, the first ten principal components of ancestry and genotyping batch.

**
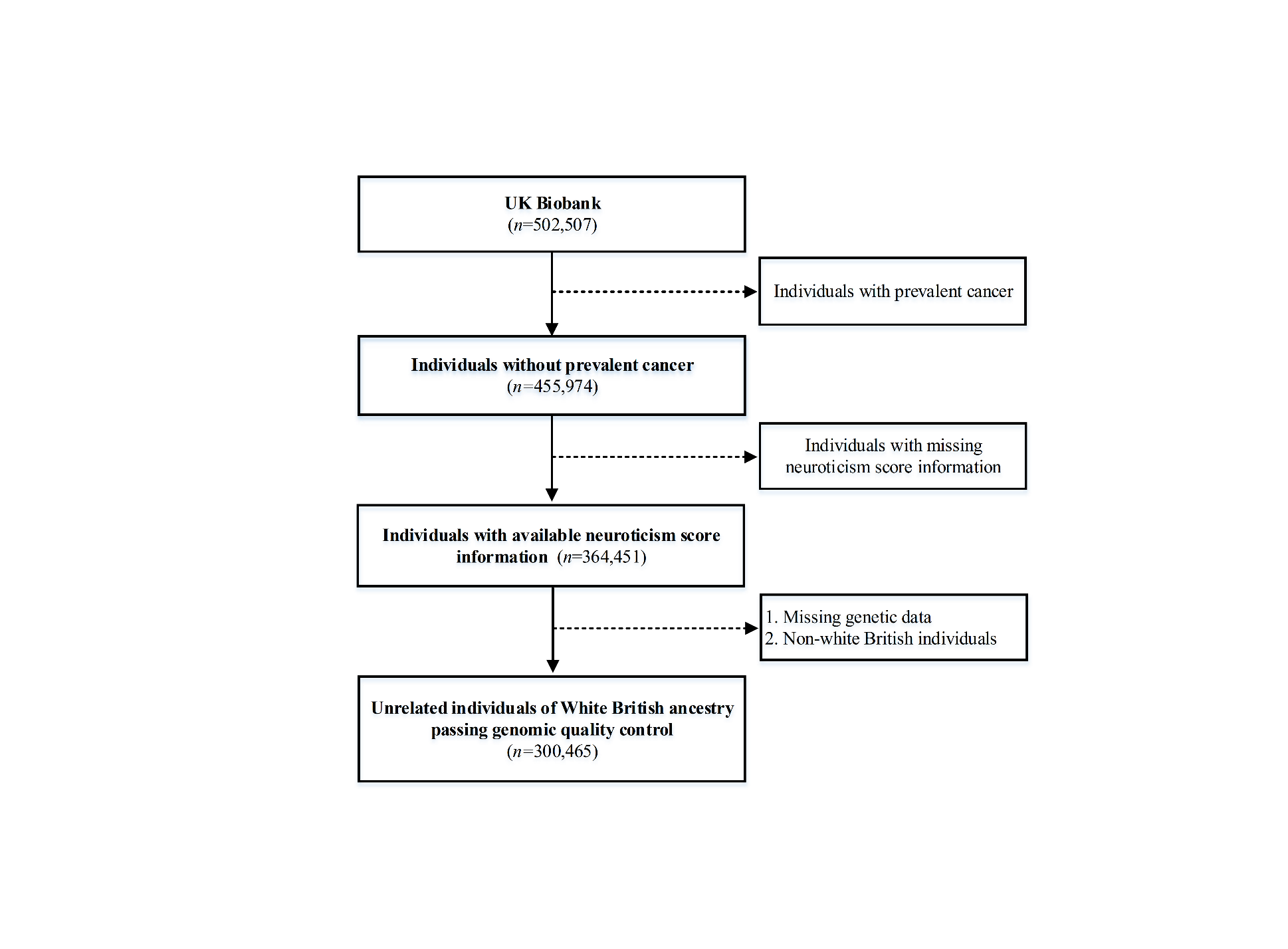
**

**Supplementary Figure S1**

**
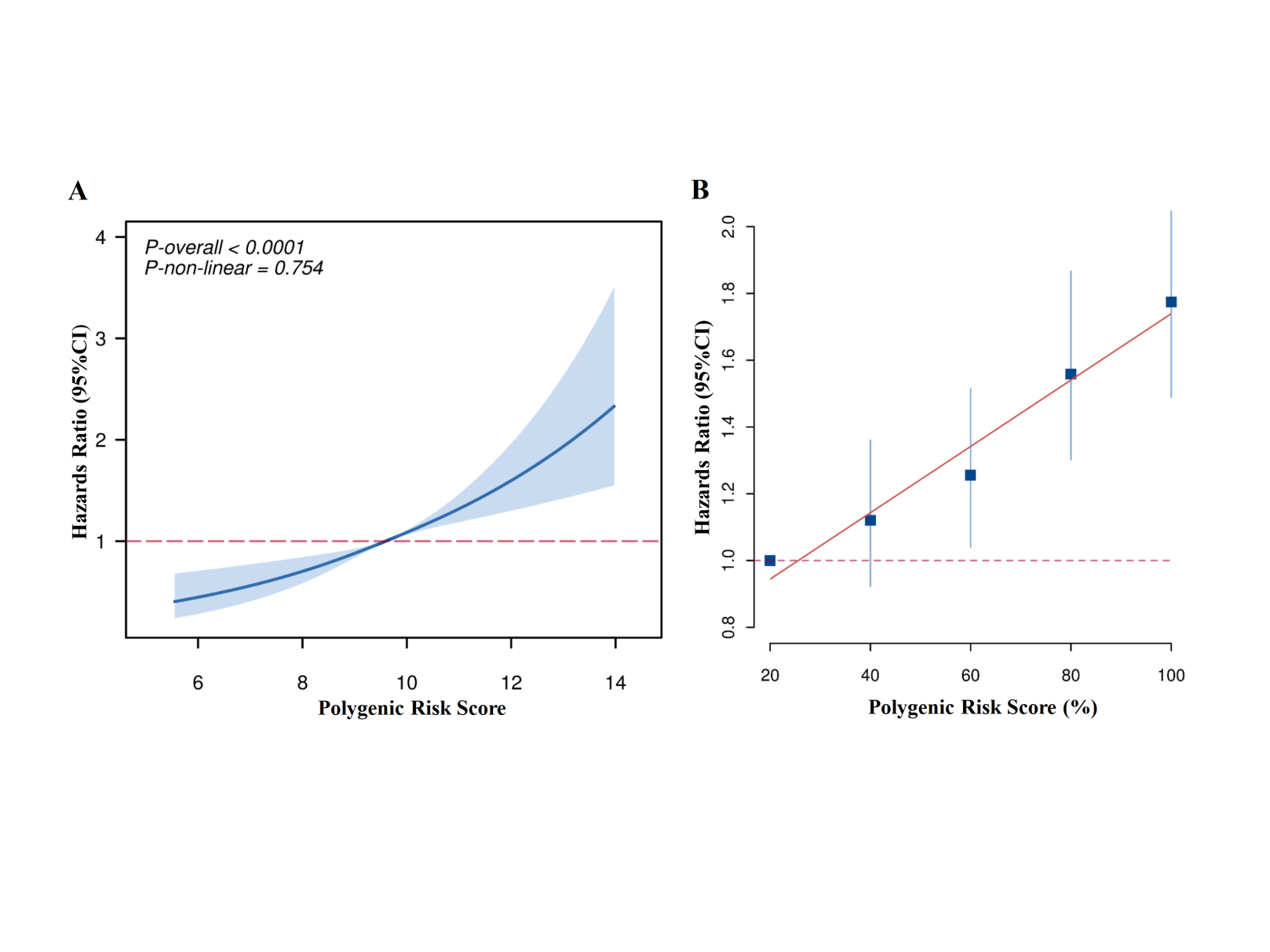
**

**Supplementary Figure S2**
